# Non-invasive blood glucose sensing by machine learning of optic fiber-based speckle pattern variation

**DOI:** 10.1117/1.JBO.27.9.097001

**Published:** 2022-09-05

**Authors:** Deep Pal, Sergey Agadarov, Yevgeny Beiderman, Yafim Beiderman, Amitesh Kumar, Zeev Zalevsky

**Affiliations:** a Bar-Ilan University, Faculty of Engineering, Ramat Gan, Israel; b Indian Institute of Technology (Indian School of Mines) Dhanbad, Department of Electronics Engineering, Dhanbad, Jharkhand, India; c Holon Institute of Technology (HIT), Faculty of Electrical Engineering, Holon, Israel

**Keywords:** classification, glucose sensor, lasers, machine learning, magneto-optics, non-invasive, optics, optical fiber sensors, speckle patterns

## Abstract

**Significance:**

The ability to perform frequent non-invasive monitoring of glucose in the bloodstream is very applicable for diabetic patients.

**Aim:**

We experimentally verified a non-invasive multimode fiber-based technique for sensing glucose concentration in the bloodstream by extracting and analyzing the collected speckle patterns.

**Approach:**

The proposed sensor consists of a laser source, digital camera, computer, multimode fiber, and alternating current (AC) generated magnetic field source. The experiments were performed using a covered (with cladding and jacket) and uncovered (without cladding and jacket) multimode fiber touching the skin under a magnetic field and without it. The subject’s finger was placed on a fiber to detect the glucose concentration. The method tracks variations in the speckle patterns due to light interaction with the bloodstream affected by blood glucose.

**Results:**

The uncovered fiber placed above the finger under the AC magnetic field (150 G) at 140 Hz was found to have a lock-in amplification role, improving the glucose detection precision. The application of the machine learning algorithms in preprocessed speckle pattern data increase glucose measurement accuracy. Classification of the speckle patterns for uncovered fiber under the AC magnetic field allowed for detection of the blood glucose with high accuracy for all tested subjects compared with other tested configurations.

**Conclusions:**

The proposed technique was theoretically analyzed and experimentally validated in this work. The results were verified by the traditional finger-prick method, which was also used for classification as a conventional reference marker of blood glucose levels. The main goal of the proposed technique was to develop a non-invasive, low-cost blood glucose sensor for easy use by humans.

## 1 Introduction

In the human body, blood sugar (glucose) is a crucial energy source for cell metabolism. Excessive blood glucose levels cause hyperglycemia, diabetes, and cardiovascular disease. Diabetes is a chronic metabolic disorder caused by insulin deficiency. There are two types of diabetes in humans. The pancreas does not create enough insulin in type I diabetes. The cells in type II diabetes do not respond to the insulin produced.^1^^,^^2^ Diabetes is a widespread disease that the World Health Organization has proclaimed a global pandemic. Diabetes is difficult to cure and has a high incidence rate and numerous complications.^3^^,^^4^ Delayed diabetes diagnosis and poor patient control are the most common causes of complications; they can lead to significant health problems and early death. As a result, diabetes has become a potentially fatal epidemic that is rapidly expanding. According to the International Diabetes Federation, 537 million adults (20 to 79 years) were living with diabetes in 2021, with a projected rise of 643 million by 2030 and 783 million by 2045. Though there are many identified diabetic patients, 1 in 2 (240 million) of the world population is undiagnosed, with 6.7 million diabetes-related deaths in 2021(1 every 5 s). According to 2021 global findings, diabetes costs the healthcare system at least 966 billion USD—an increase of 316% worldwide over the last 15 years.^5^ As a result, measuring blood sugar (glucose concentration) is crucial for diabetes control.

Blood sugar monitoring devices are a significant field in diabetes research, potentially enhancing the lives of more than 537 million people worldwide. Although there is currently no treatment for diabetes, a suitable and invasive blood glucose monitoring device can help patients avoid diabetic complications such as kidney damage, congenital disabilities, heart disease, stroke, and neuropathy.^1^^,^^6^ The most common method for diagnosing diabetes is to monitor blood glucose levels. A cost-effective electrochemical biosensor is used as a commercial blood glucose monitoring device. The sensor relies on the most reliable approach for patient glucose self-monitoring, which is the classic finger-prick method using glucose strips and a meter. However, a blood sample requires piercing the patient’s fingertips with lancet instruments, which is uncomfortable and inconvenient when frequent testing is required. The finger-prick method is well known for producing precise glucose readings, but repeated skin puncture is inconvenient and can lead to infections. Recently developed devices for continuous blood glucose monitoring incorporate advances in microelectronics, implantable materials, and wireless technology, but most are invasive or minimally invasive.^1^ As a result, one of medicine’s most important objectives is to develop a non-invasive method for monitoring blood glucose levels.^3^^,^^4^^,^^7^^,^^8^

The research for non-invasive glucose monitoring technologies began in 1975 and continues. The demand for a cost-effective, compact, painless, convenient, non-invasive device to relieve pain by monitoring glucose levels without pricking the skin barrier has increased.^9^^,^^10^ In recent years, non-invasive devices for measuring human blood glucose levels have been developed; these include the MediWise GlucoWise, Nemaura Medical SugerBEAT, Cnoga Medical (Combo Glucometer), Integrity Applications, C8 MediSensors, and OrSense (NBM-200G). The devices use different optical, thermal, transdermal (electrochemical), and microwave methods for non-invasive glucose monitoring.^11^ Omer et al.^12^ proposed a low-cost mm-wave radar to detect glucose concentration through correlation using reflected mm-wave readings. Haxha and Jhoja^6^ used near-infrared (NIR) at the wavelength ranging from 750 to 2500 nm on fingertips to detect blood glucose. Guevara et al.^13^ used NIR spectroscopy with electrical impedance spectroscopy for glucose monitoring. In Ref. 14, Shokrekhodaei et al. used non-invasive optical sensors with multiple wavelength measurements for glucose monitoring using machine learning (ML) algorithms*.* None of the current non-invasive technologies have demonstrated the long-term accuracy required to replace finger prick procedures. They have all failed to run for an extended period.^4^^,^^7^^,^^15^ The indirect nature of the measurement and the unavoidable calibration procedure are the most significant hurdles in developing non-invasive glucose measurement sensors. They also necessitate improving the signal-to-noise ratio and sensitivity, evaluating analytical performance, developing accurate blood glucose measurement methodologies, and reducing measurement time. As a result, a more reliable glucose measuring device is required.^11^ One of the promising noninvasive methods of blood glucose evaluation is related to the use of multimode optic fibers to transmit laser-induced light. Interference between the coherent multimode light rays inside the fiber creates primary speckle patterns at the fiber exit. Interaction between the optic fiber and the skin creates speckle pattern variation, which can be recorded and processed by correlation techniques. Such a sensor could be used to create a smart cloth for heartbeat and respiration monitoring.^16^

This paper proposes a new non-invasive blood glucose testing technology that generates and analyzes primary speckle patterns formed by light rays interacting with the skin inside an uncovered multimode optic fiber. Our multimode fiber (MMF)-based optic sensor is developed for non-invasive glucose monitoring systems and can be used to obtain higher sensitivity^15^^,^^17^^–^^19^ The experiments involve the subjects’ finger placed on a fiber under normal conditions and under the influence of a magnetic field while the tested subject had varying blood glucose levels. It was found that the magnetic field improves the sensitivity and accuracy of the blood glucose concentration measurements by an uncovered multimode optic fiber sensor. The speckle pattern variation can be evaluated by currently available techniques such as statistical analysis, morphological image processing, and correlation metrics. Due to the difficulties of non-invasive glucose measurements and noisy recordings, an advanced data analysis technique is required to improve glucose level detection accuracy. The innovative aspect of this study is the employment of uncovered optic fiber (with cladding removed) touching the skin subjected to an AC-generated magnetic field to filter the glucose signal from noise. In addition, the uncovered MMF enhances the observability of the magneto-optic effect. The ML algorithms capable of handling higher-dimensional data that can learn and classify different blood glucose levels are used.^14^^,^^20^

## 2 Material and Methods

### 2.1 Background of the Proposed Technique

**Fig. 1:**
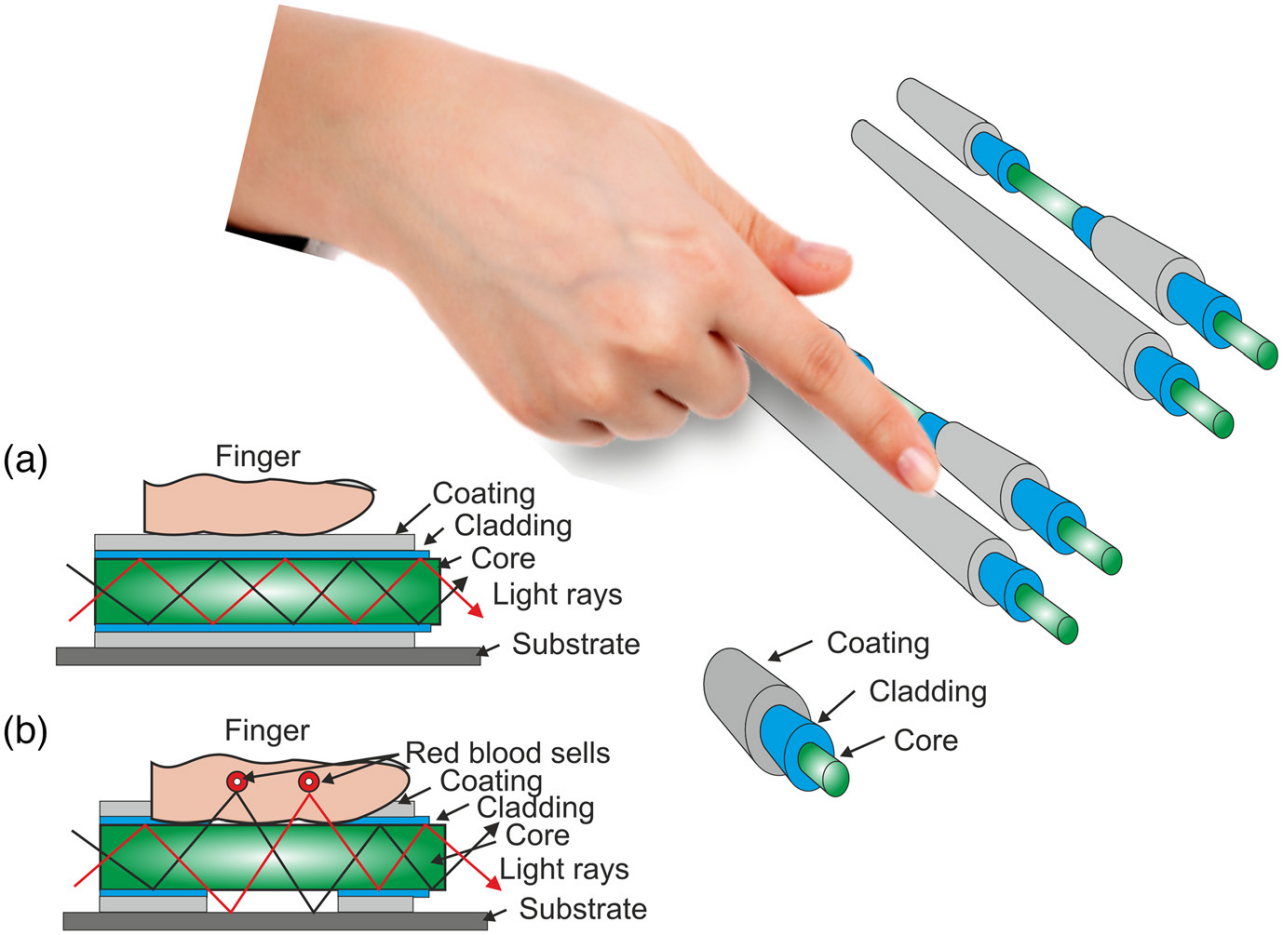
Multimode fibers (a) covered fiber and (b) partially uncovered fiber with removed coating and cladding.

Blood consists of both liquid and solid components. Plasma is a liquid composed of water, ions, and protein that makes up half of the blood. Red blood cells (RBC), white blood cells, and platelets make up the solid part of blood. Hemoglobin is a protein molecule found in RBC. The actual part of the blood that glucose attaches to is hemoglobin. Hemoglobin’s primary goal is to deliver oxygen to tissues and organs from the lungs. After absorption by the gastrointestinal system, nutrient components from food reach the bloodstream, and if glucose is present, glucose is also delivered.^21^ Hemoglobin is an iron-containing protein that is a para-magnetic element influenced by the magnetic fields.^22^^,^^23^ The proposed technique is based on the light tissue interaction induced on the sensing fiber considering the effect under the magnetic field and without it. In the case of covered fiber, the sensing is due to skin vibration as there is no light leakage, as shown in Fig. 1(a). In the case of uncovered fiber, the cladding and jacket (coating) are removed, which creates an evanescent wave (EW) to achieve direct interaction of light with the subject’s finger. Hence, the light leakage from the uncovered fiber is highly sensitive and can penetrate the skin barrier detecting the blood plasma glucose and the iron-containing protein—hemoglobin with the attached glucose transported to the body cells [Fig. 1(b)].

The intensity of EW decays exponentially with the increase in core and subject finger distance, known as penetration depth:

$$d_{p}=\frac{\lambda }{2\pi (n_{\mathrm{eff}}^{2}-n_{s}^{2}{)}^{1/2}},$$ (1)

where λ is the light’s wavelength, $n_{s}$ is the refractive index of the surrounding medium, and $n_{\mathrm{eff}}$ is the optical fiber’s effective refractive index.^24^^,^^25^ The interaction of EWs with the surrounding tissue can occur with the uncovered fiber. As a result, the output power fluctuates depending on the optical characteristic of the sensing medium. In an MMF with the cladding removed, the output power is computed as

$$P_{\mathrm{trans}}=P_{0}\exp(-\gamma_{\mathrm{eff}}L/D),$$ (2)

where $P_{0}$ represents the reference power (without cladding modification), L is the active length (sensing region) of the fiber, D represents the core diameter, and $y_{\text{eff}}$ is the EW absorption coefficient, which depends on both the Fresnel transmission coefficient at the interface of the core and modulated cladding and the frequency of guided ray reflections per unit length of the optical fiber (sensing region).^26^

The induced changes in the sensing fiber (covered or uncovered) will result in the modulation of the refractive index, spectral absorption, phase, and polarization of the photons of light traveling along the fiber and interacting.^27^ This modulation can be detected by recording changes in a random self-interference image called speckle pattern. Speckle patterns allow us to follow changes in the phase and amplitude of light inside the fiber and record the primary speckle pattern images from the fiber’s end.

When coherent light passes through an MMF, the propagating light modes, all considered to be equally excited, interfere and produce a speckle pattern. The fiber far-field speckle distribution $A_{0}$ is the superposition of all of the modes’ amplitudes.^19^

$$A_{0}(x,y)=\sum_{m=0}^{M}a_{om}(x,y)e^{j\varphi_{om}(x,y)},$$ (3)

where M is the number of light modes inside the fiber, which is related to the fiber diameter and coherent light wavelength, and $a_{0\;m}(x,y)$ and $\varphi_{0\;m}(x,y)$ are the amplitude and phase of mode m of a pixel (x,y), respectively. The far-field speckle pattern intensity I(x,y), captured by a defocused camera from the fiber exit, is described as follows:

$$I(x,y)=|A_{0}(x,y){|}^{2}=\sum_{m=0}^{M}\sum_{n=0}^{M}a_{0\;m}(x,y)a_{on}(x,y)e^{\partial (\varphi_{om}(x,y)-\varphi_{on}(x,y))},$$ (4)

where $a_{0n}(x,y)$ and $\varphi_{0n}(x,y)$ are the amplitude and phase of mode n of pixel (x,y), respectively.^16^

Under the influence of a magnetic field, the light-tissue interaction of an uncovered MMF induces perturbations in the propagation medium at the inferred frequency of each mode. The interaction has distinct effects on the modes; amplitude and phase deviate depending on the mode index. Tracking and analyzing speckle patterns of the recorded images when the fiber interacts with the tissue can help to differentiate between blood glucose levels using a correlation-based algorithm.^16^^,^^19^

### 2.2 Analysis of Recorded Speckle Patterns

Defocused speckle imaging is an optical method in which a defocused camera records the scattered interference of the primary speckle pattern that characterizes the modulation in the sensing fiber or secondary—reflected from a surface illuminated by a laser beam.^8^^,^^10^^,^^20^ Speckle motion tracking enables the detection of disturbances in the sensing fiber from a distance.^17^

**Fig. 2:**
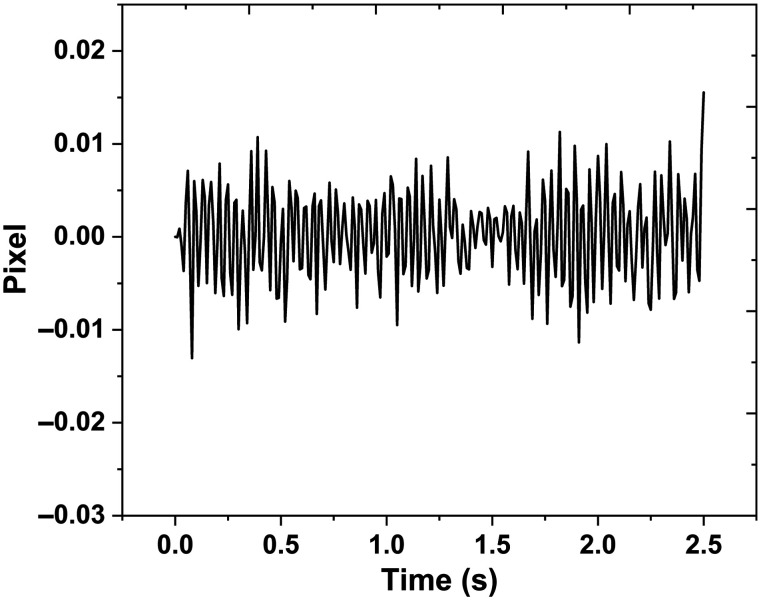
Experimental results of the blood glucose sensing of subject 1.

The multimode fibers interacting with the tissue under different glucose levels change the speckle patterns. In the case of no magnetic field being applied, the variation is negligible for the covered and uncovered fibers. Under an alternating current (AC) generated magnetic field affecting the skin, a covered fiber restricting light-tissue interaction has a negligible blood glucose sensing effect. However, an uncovered fiber interacting with the skin affects the speckle pattern, as the leaking light changes polarization under Faraday’s effect and reflects from the iron-containing hemoglobin affected by the magnetic field. The method can classify blood glucose levels after removing noise components and inferring data at the applied magnetic field frequency. First, the cross correlation of the speckle pattern images was used to determine its displacement across the x and y axes of the image, given that the two images differ only by an unknown shift. When the images match, the value of the cross-correlation function is maximized.^16^^,^^19^ Speckle pattern correlation is used to analyze positional shifting between two adjacent speckle patterns to determine each pixel’s temporal correlation. The two speckle patterns I and $I^{\prime }$ are usually divided into a set of sub-images. The cross-correlation algorithm tracks the movement of a number of speckles acting together as a sub-image. The correlation function is calculated for each pair of corresponding sub-images, and the respective displacement is derived from the maximum position. Sub-image A in image I is allowed to sweep over image $I^{\prime }$. When the area with the highest statistical agreement (cross-correlation) is found, it is labeled as sub-image $A^{\prime }$ and is considered to correspond to sub-image A. The discrete cross-correlation between A and $A^{\prime }$ is calculated as follows:^28^

$$R_{AA^{l}}(d_{x},d_{y})=\frac{1}{NM}\overset{N-1}{\underset{i=0}{\sum }}\overset{M-1}{\underset{j=0}{\sum }}A(i,j)A^{l}(i+d_{x},j+d_{y}),$$ (5)

where $d_{x}$ and $d_{y}$ are displacements in the x and y directions, respectively. The movement over the surface position A to $A^{\prime }$ is found by the position of the correlation peak and is given by the displacement vector of the midpoint of sub-image A. These calculations are done for all sub-images of I until a displacement field of the whole surface is obtained, meaning that the movement is determined in two directions, x and y. The height of the correlation peak indicates how similar the cross-correlated sub-images are and hence yields a value of the accuracy of the measurement.^28^ Figure 2 shows the correlation between images for one subject at a particular glucose level instance, representing peaks for the particular glucose level. It shows the temporal changes in the correlation peak’s location in pixel units for a single recording of an individual before data preprocessing. We extracted the position of the correlation peak and plotted its time-varying position, with the amplitude denoting the shift in the position of the correlation peak in pixel units of the camera (Fig. 2). All relative movements are cumulatively summarized for extracting the total movement vector. The process of recording speckle images for both covered and uncovered fibers for all subjects at different glucose levels is shown in Fig. 4.

### 2.3 Machine Learning Analysis

ML applications in optical sensors have grown in prominence in recent years, particularly in monitoring and improving the detection accuracy of optical sensors for enhanced performance. Supervised ML algorithms process sample data, referred to as training data, to make predictions on unknown data without being explicitly programmed to achieve the intended goal.^14^ We used several classification algorithms to classify glucose levels. The Naïve Bayes algorithm gives the best results compared with other algorithms such as support-vector machines (SVMs), neural networks, and K-nearest neighbors (KNN). The Naïve Bayes classifier works on the idea of conditional probability, with a strong assumption that the attributes are conditionally independent given the class. Using our data, Naïve Bayes provides a mechanism for estimating the posterior probability P(y|x) of each class y given a predictor x. The Naïve Bayes algorithm has excellent computational efficiency, low variance, and direct posterior probability prediction incremental learning. The essential aspect is that it is somewhat noise-resistant because all predictions are made using attributes.^29^^,^^30^

By assuming that attributes are conditionally independent based on the given class, the algorithm first estimates the densities of the predictors within each class.

P(y|x)=P(y)P(x|y)/P(x), (6)

where P(y) is the prior probability of the class, P(x|y) is the probability of the predictor of the particular class (y), and P(x)is the prior probability of the predictor. Eqution (6) for the model posterior probabilities according to the Bayes rule is also described as,^31^ i.e., for all class indexes k=1,…,K,

P^(y=k|x1,…,xp)=π(y=k)∏j=1pP(xj|y=k)∑k=1Kπ(y=k)∏j=1pP(xj|y=k), (7)

where π(y=k) is the prior probability that a class index is k,y is the random variable corresponding to the class index of an observation, and $x_{1},\ldots ,x_{p}$ are the random predictors of an observation. Finally, the algorithm assigns each observation to the class providing the highest posterior probability after calculating the posterior probabilities for each class.^31^

**Fig. 3:**
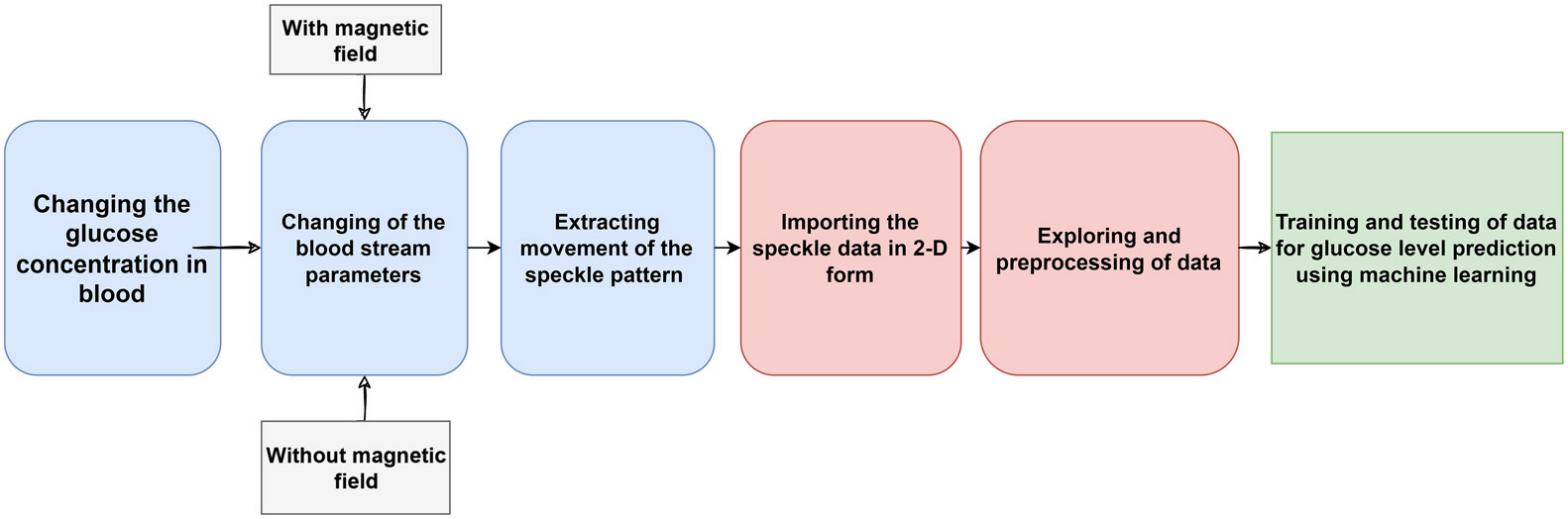
Blood glucose detection flow chart.

The above-mentioned process of recording the speckle patterns and preprocessing the recorded data for blood glucose level classification using ML algorithms is shown in Fig. 3.

### 2.4 Experimental Setup

**Fig. 4:**
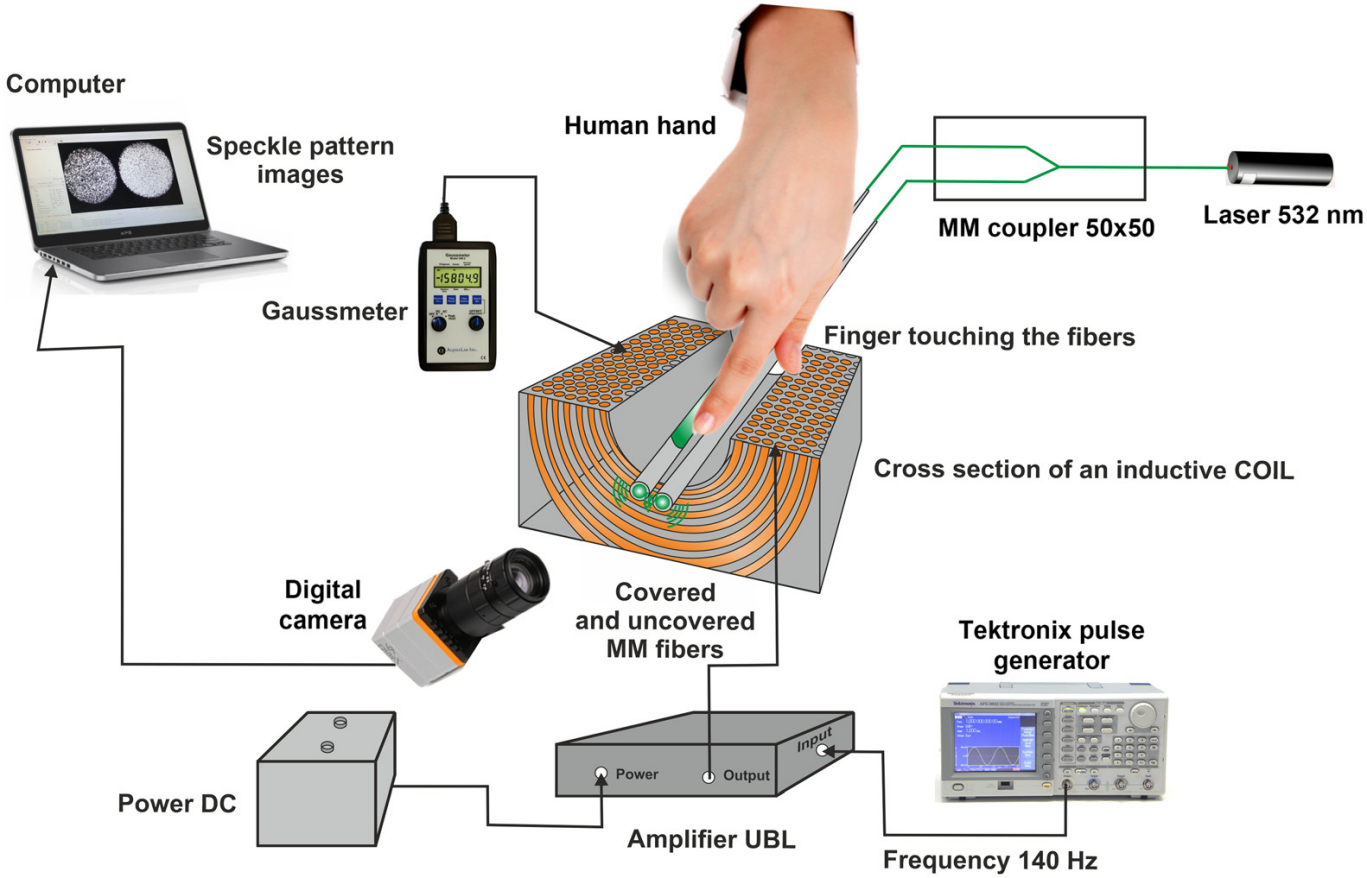
Schematic diagram of the experimental setup.

The experimental setup schematic diagram is shown in Fig. 4.

**Fig. 5:**
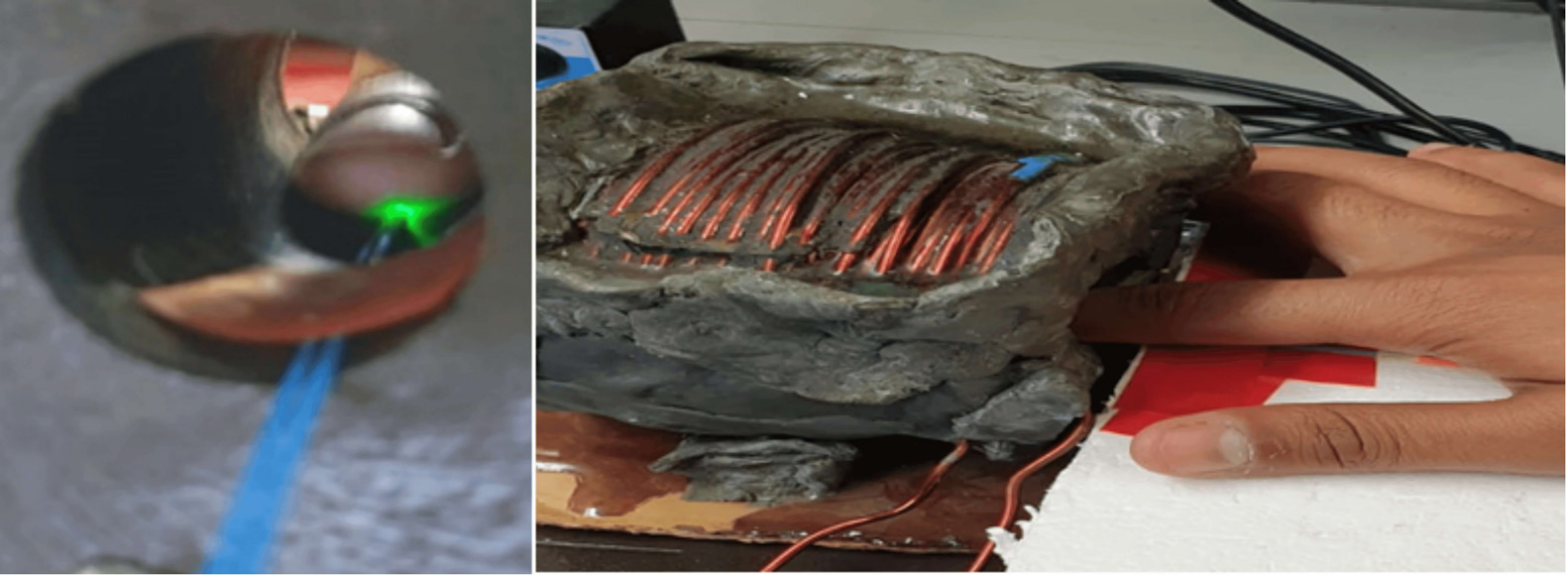
Experimental setup element.

The sensors configuration consisted of a green laser (532 nm), defocused Basler camera, magnetic field inductor, and multimode optic fibers (covered and uncovered) with a core diameter of 400±8  μm, cladding diameter of 425±10  μm, and coating diameter of 730±30  μm. The laser was connected to the two fibers through a light splitter. The fibers passed along the solenoid axis. The subject’s finger, touching the fibers, was inserted into the solenoid. The magnetic field, created by the AC and passing the solenoid, influenced the inserted finger. The camera captured speckle pattern images generated by a laser beam exiting multimode fibers. The camera was connected to a computer that processed the speckle patterns captured from both fibers. Figure 5 shows the setup element, containing the MM fibers passing through the solenoid and the human finger touching the covered and uncovered fibers.

In this experiment, the fiber sensors generated primary speckle patterns affected by the interaction with the subject’s finger area for both (covered and uncovered) fibers without magnetic field induction. In addition, each subject was tested under an AC-induced magnetic field of about 150 G (0.015 T) inferred at a particular frequency (140 Hz). The magnetic field was generated by the electric current passing through the electromagnet to generate a magneto-optic effect on the blood to classify glucose concentrations. The current was generated by a Tektronix pulse generator and amplified using a standard JBL audio amplifier powered by a 12 V battery. The intensity of the magnetic field was measured using the standard GM2 Gauss meter.

## 3 Results

Our experiment gathered data from six healthy subjects aged 24, 25, 27, 28, 60, and 75. First, the subject’s blood glucose was measured by the traditional finger-prick method as a base-true glucose label. After the traditional test, the subject was shortly tested with our optical setup for different glucose levels. The measurements were done in the morning after 12 h of fasting to fix the low glucose level and then after a meal and for subsequent time intervals to get the variation in glucose levels. The participant inserted the tested finger inside the solenoid to touch and cover the fiber, passing the solenoid. Before each recording, we checked the magnetic field strength, and the finger was re-position to avoid overfitting. The speckle pattern recordings were repeated five consecutive times. Each recording received the same reference, and the set was sub-divided for training and validation. The reference measurements for the tested subjects are presented in ascending order in Table 1.

**Table 1:** Reference blood glucose measurements for the tested subjects.

Sample number	1	2	3	4	5	6	7	8	9	10	11	12	13	14
Glucose (mg/dl)	86	93	97	103	105	110	111	115	119	126	132	147	153	170

**Fig. 6:**
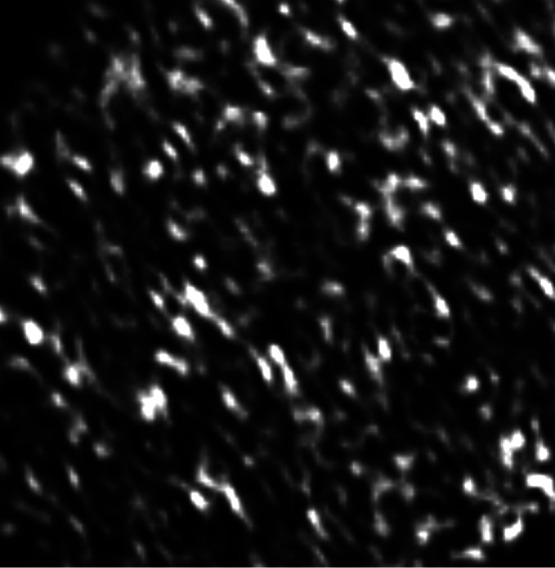
Picture of speckle pattern at the output of the multimode fiber.

**Fig. 7:**
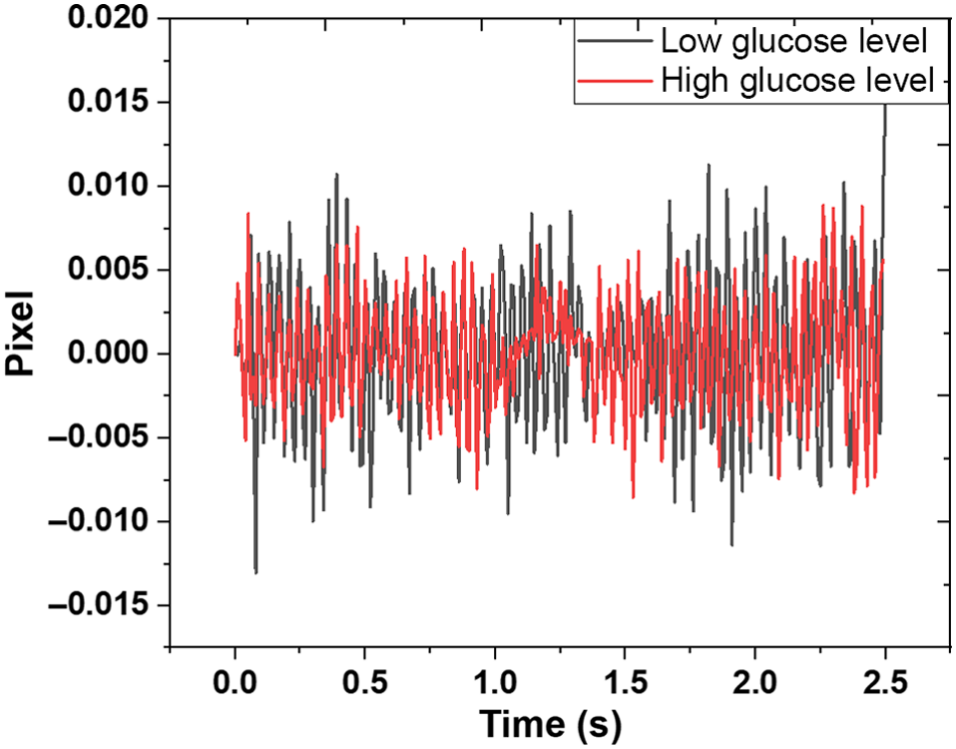
Data used to train the algorithm to find the variation in different glucose levels for a magnetic field.

The Basler camera recorded the change in speckle pattern captured from the multimode fibers exit. We employed both covered and uncovered fibers to see how changes in glucose concentrations affect the change of speckle patterns caused by light and the finger tissue interaction. Both fibers were tested without a magnetic field and under a magnetic field, generating a magneto-optic effect. An example of the acquired image of 128*128 pixels in size is shown in Fig. 6. Each speckle image was correlated to the next frame using MATLAB code throughout the preprocessing data phase. For each image, the correlation between the current and reference frames was calculated and averaged over time. Figure 7 shows the sample data of time-averaged signals for different configurations used. The variation from min to max of the peak correlation value in time exhibits a lower value for high glucose. The high value to low glucose may be due to the effect of glucose on the RBC. Under the influence of the AC induced magnetic field, the RBC containing iron oscillate. When the blood glucose levels are high, an increased number of glucose molecules attach to the hemoglobin, which may result in higher inertia, causing a lower value in speckle variation for high glucose levels.

**Fig. 8:**
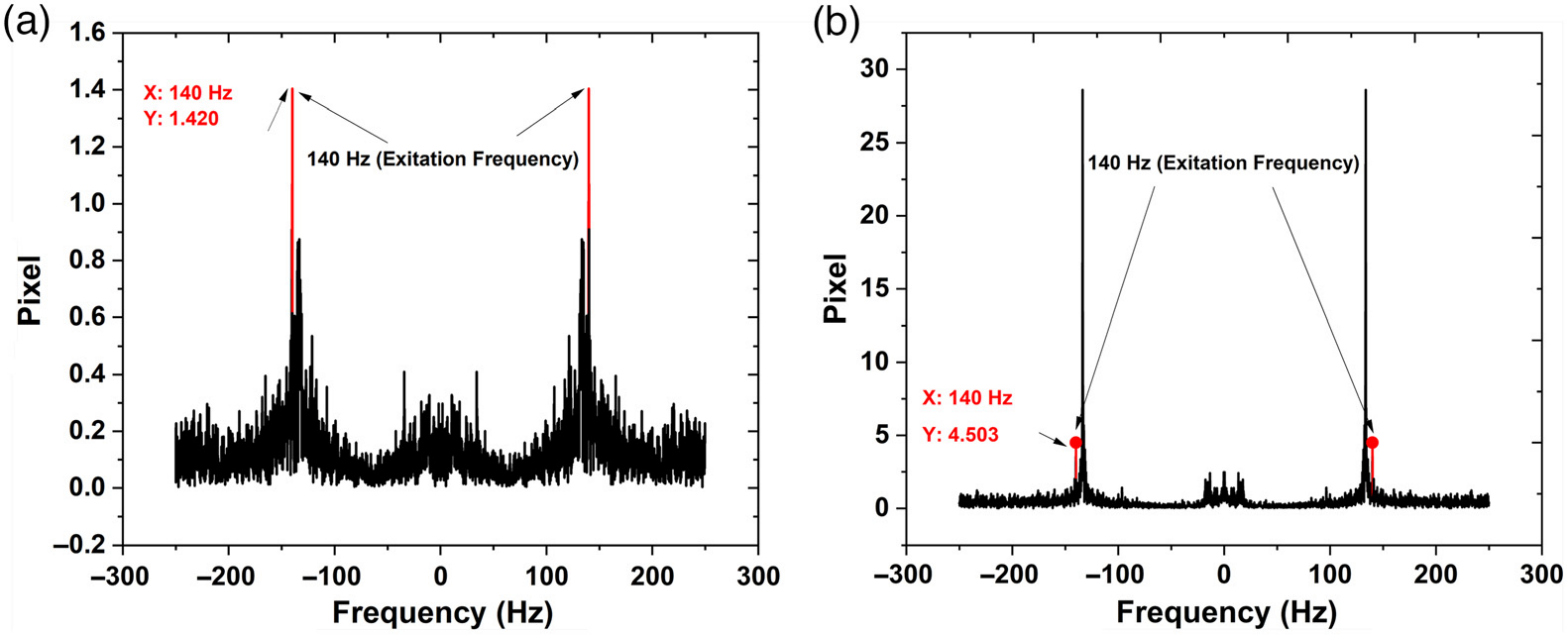
The frequency (spectral) response, under magnetics excitation frequency 140 Hz. (a) Covered fiber and (b) uncovered fiber.

**Fig. 9:**
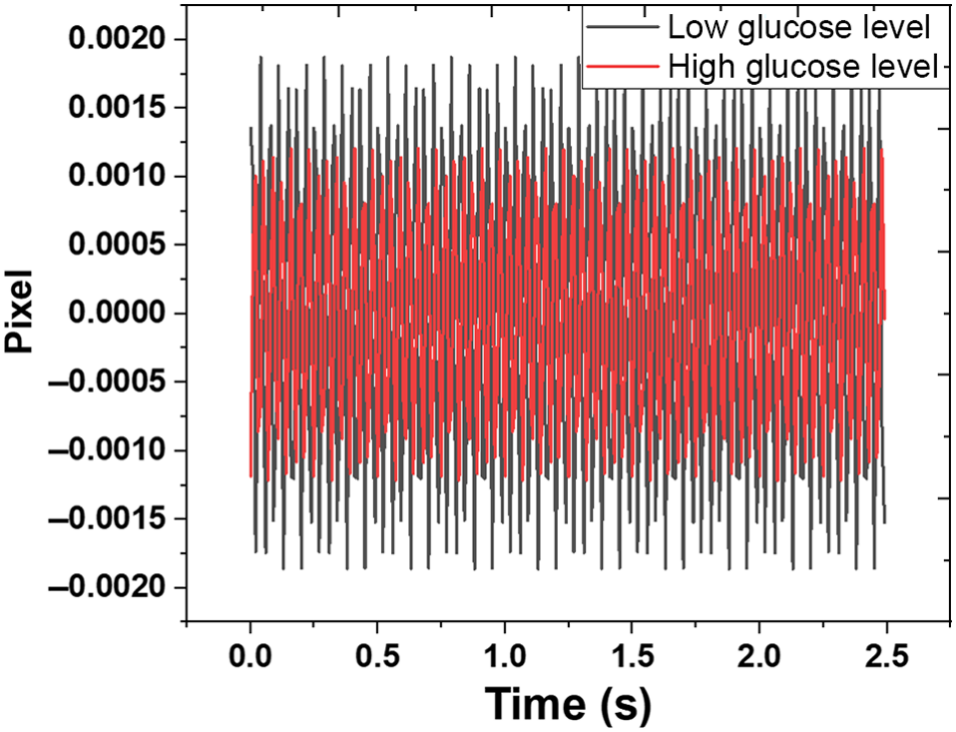
Sample data used to train the algorithm to find the variation in different glucose levels for magnetic field inferred at 140 Hz.

The data from the tests without a magnetic field was directly used for ML. In contrast, the data recorded under a magnetic field generated at 140 Hz, was inferred by converting the data into the frequency domain using fast Fourier transform (FFT). The fluctuation in the speckle pattern under the magnetic field at 140 Hz was obtained by selecting our frequency of interest (140 Hz) from the FFT signal and removing all other frequency components using Matlab. To retrieve data containing fluctuation only at 140 Hz, we used inverse FFT (IFFT) to get back our time series data. The retrieved IFFT data contains signals at a particular glucose level. In Fig. 8(a), the FFT of the covered fiber, which depicts the change due to skin vibration, is dominant and less effective at 140 Hz because it is not related to glucose level as there is no light leakage. It only captures the data due to vibration at 140 Hz. In the case of uncovered fiber, there is a four times increase in the amplitude of the FFT signal at 140 Hz, which is related to glucose level change due to light tissue interaction, as shown in Fig. 8(b). The acquired IFFT signal of uncovered fiber is free from external noise and can be used to classify different glucose levels, as shown in Fig. 9. This data are normalized and used to train an ML system to distinguish between glucose levels. We analyzed the results for the tests without the magnetic field, for an AC magnetic field without data filtering, and for an AC magnetic field inferred at 140 Hz to differentiate between performances of all configurations used.

We used the Naïve Bayes algorithm for the ML of the data. The Naïve Bayes algorithm was optimized and trained for classification. The algorithm was tuned for the best hyper parameters by grid search optimization to find the optimal conditions. The hyper parameter optimization was done to increase the performance of the classification. The triangle Kernal type gives better results than kernel types, such as Gaussian, box, and Epanechnikov. Bayesian optimization was used as an optimizer. We applied a common cross-validation technique, K-fold (five fold) cross-validation, to prevent the algorithm from overfitting. The data were divided into five randomly chosen subsets (or folds) of nearly similar size. The model trained with the remaining subsets was validated using one subset. This step was repeated five times to ensure that each subset was validated exactly once.

**Fig. 10:**
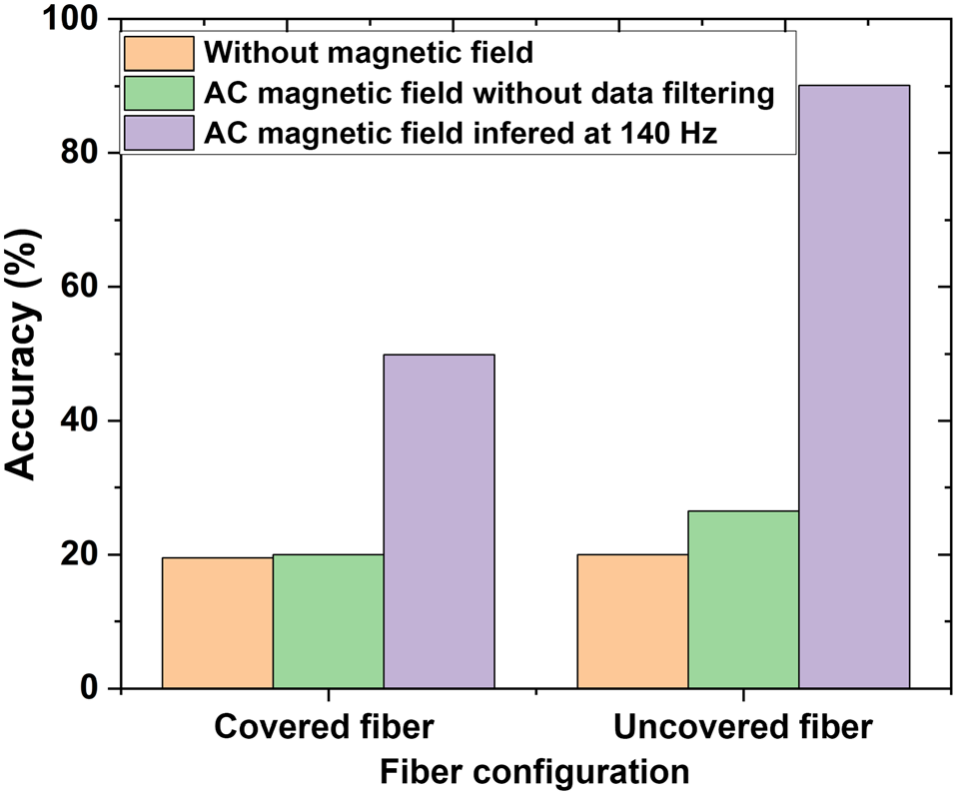
Classification accuracy of the blood glucose level for different configurations.

During the testing, 14 distinct glucose levels were selected from the recordings. The 2D preprocessed speckle pattern data for the selected glucose levels was sub-divided and used as ML data input. We selected 60% of the data to train the algorithm as the first step of ML processing. For testing, 40% of each glucose level’s sample data was initially picked at random for testing the trained Naïve Bayes algorithms for classification. After classifying the glucose data for all sensor configurations, we found that the uncovered fiber gives the best results when inferred at a 140-Hz AC-generated magnetic field. The higher accuracy is due to lock-in amplification, which improves the detection by considering the magneto-optic effect. However, the classification accuracy is very low in the case of only using the AC magnetic field without selecting data at an inferred frequency, as seen in Table 2. The uncovered fiber is only sensitive under the magnetic field due to the magneto-optic effect. Selecting data only at an inferred frequency allows for filtering noise and increases glucose sensing accuracy. The model accuracy is presented in Table 2 for both covered and uncovered fibers. Figure 10 shows the tabular representation of accuracy for all configurations analyzed.

**Table 2:** ML algorithm processing results.

COVERED_FIBER (400 μm)		UNCOVERED_FIBER (400 μm)	
Configuration	Accuracy (%)	Configuration	Accuracy (%)
Without magnetic field	19.5	Without magnetic field	20
AC magnetic field without data filtering	20	AC magnetic field without data filtering	26.5
AC magnetic field Inferred at 140 Hz	49.9	AC magnetic field inferred at 140 Hz	90.1

**Fig. 11:**
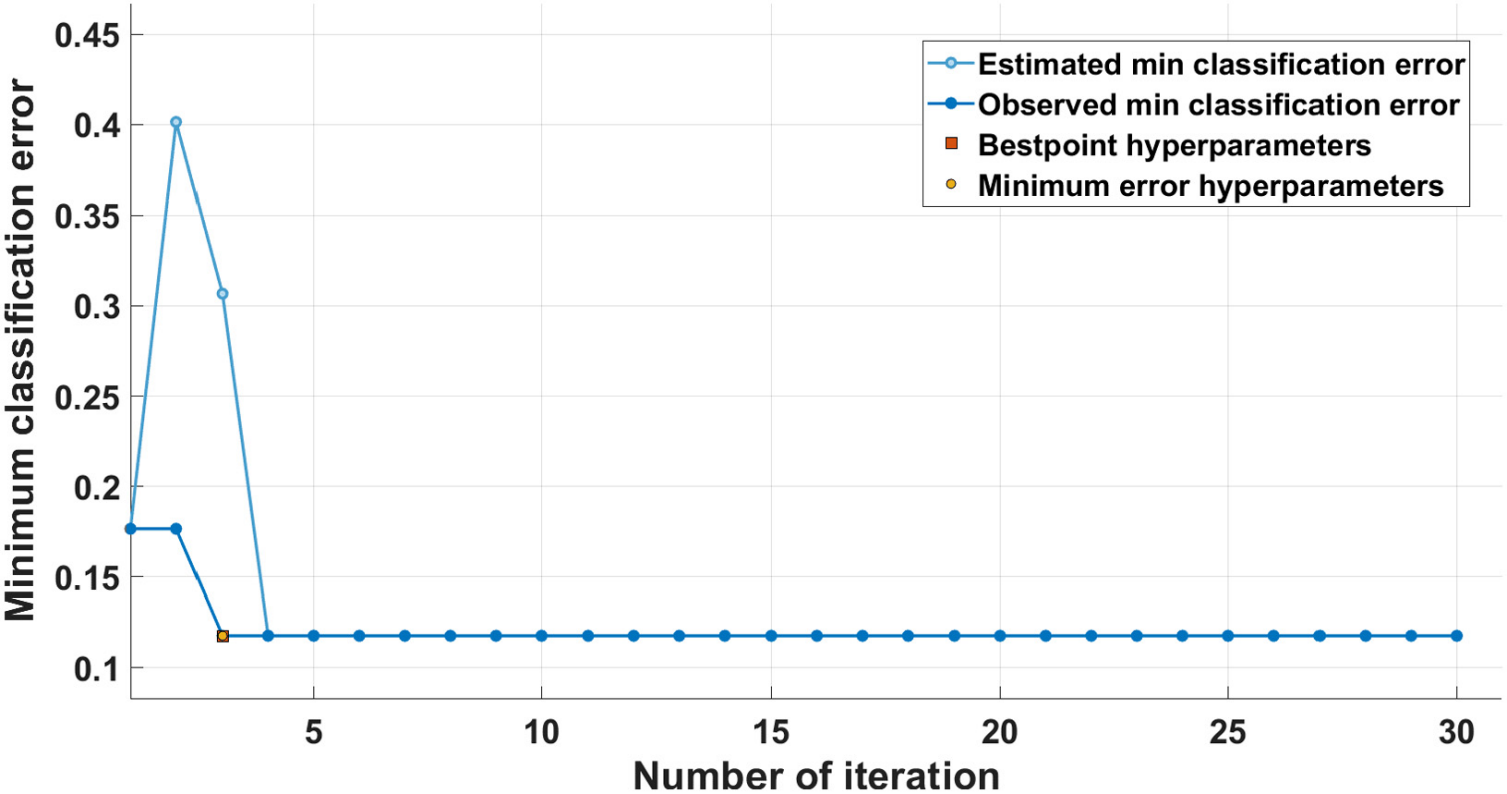
Training process of the optimized Naïve Bayes classification algorithm.

**Fig. 12:**
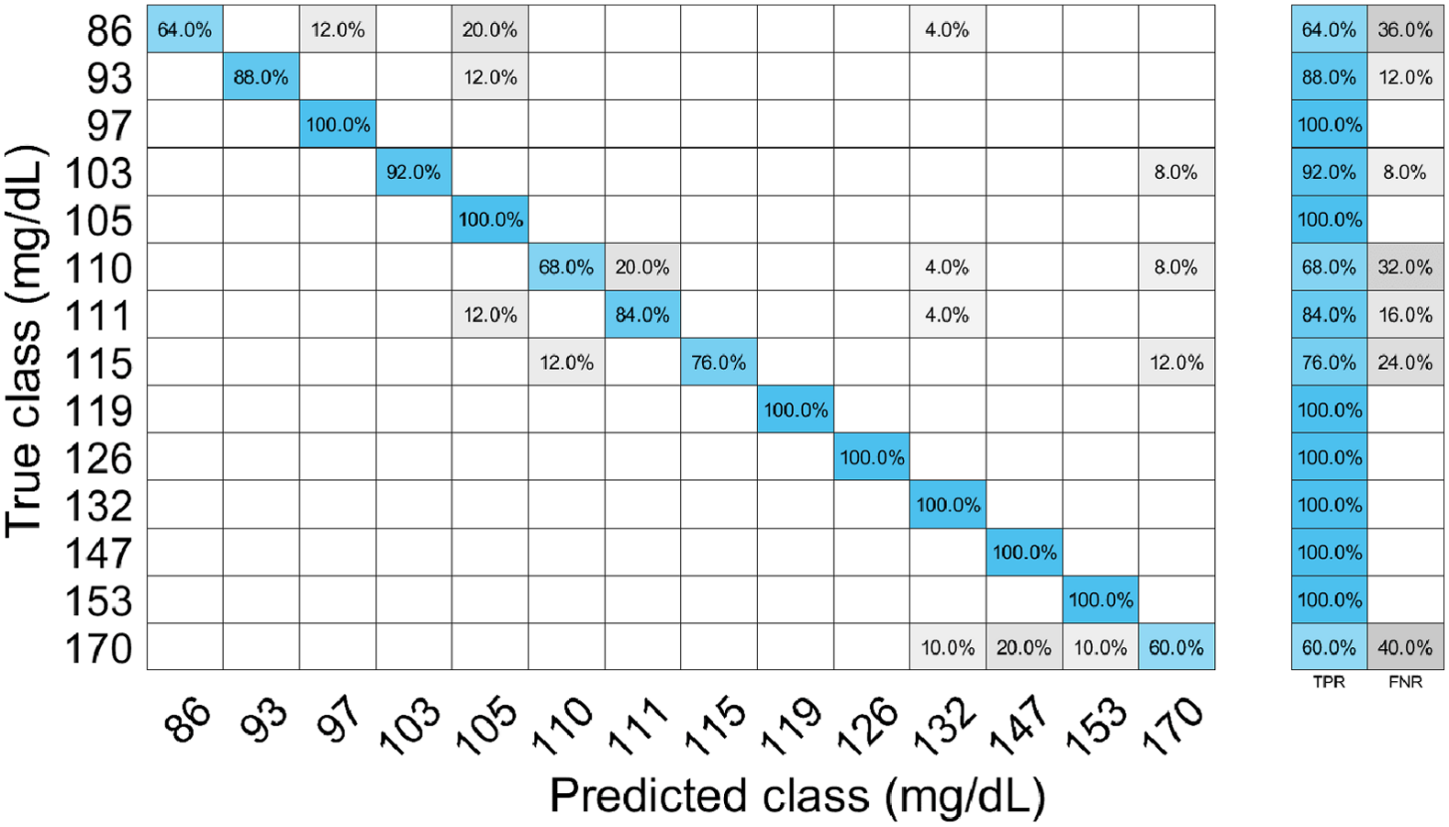
Confusion matrix.

As shown in Fig. 10, there is a significant improvement in the blood glucose detection accuracy under the magnetic field at a lock-in frequency of 140 Hz for uncovered fiber compared with other configurations. The training process of our best configuration for the minimum classification error is shown in Fig. 11. The accuracy of the trained model was verified using the test data; see the confusion matrix, Fig. 12, which depicts the classification accuracy for different glucose levels. The performance metrics calculated using the confusion matrix of different glucose levels are provided in Table 3. Thus, under the influence of the AC-generated magnetic field, the selectivity for changes in glucose levels can be increased due to removing the noise from the recordings using a magneto-optic effect at a fixed frequency.

**Table 3:** Performance metrics of classification.

Class	86	93	97	103	105	110	111	115	119	126	132	147	153	170
Accuracy (%)	97.35	99.12	99.12	99.41	96.76	96.76	97.35	98.24	100	100	98.53	98.82	99.41	95.59
Precision	0.64	0.88	1	0.92	1	0.68	0.84	0.76	1	1	1	1	1	0.6
Recall	1	1	0.87	1	0.76	0.85	0.81	1	1	1	0.8	0.86	0.93	0.63
F1 score	0.78	0.94	0.93	0.96	0.86	0.76	0.82	0.86	1	1	0.89	0.93	0.96	0.62

## 4 Discussion

We have experimentally demonstrated the possibility of blood glucose sensing by a laser-induced uncovered MM Fiber touching the tissue inferred at a particular magnetic field excitation frequency. This finding has practical implications for glucose sensing because fiber-based sensors are relatively easy to deploy and simple to use.

We want to point out that we chose the 140-Hz frequency because it allowed us to keep a stable magnetic field through the experiment and gave a good response. Due to the limitation of the setup, the 150 G was the stable magnetic field that was found responsive. The optimization of frequency and the magnetic field is required in future analyses, which can increase the magneto-optic effect resulting in higher accuracy of our method. Studying the effect of different glucose types, such as glycated hemoglobin (HbA1c), a stable and accurate biomarker for diabetes diagnostics, is also essential and should be addressed. The in-depth study using a more advanced optical setup and a more accurate reference device (spectrophotometer) can be incorporated to analyze different external effects (tissue interference) on optical properties, improving the sensor performance.

Using modified and structured optical fibers can further improve the accuracy and sensitivity of glucose measurements. Incorporating the MM sensor into a smart cloth for simultaneous detection of the vital body responses, including blood glucose, should also be evaluated.

Several technical challenges must be focused on to ensure the increase in operational stability and repeatability of the measurements, mainly to analyze different external inferences affecting the measurements including skin types. The efficiency and accuracy of blood glucose detection can also be further improved by advanced computational analysis.

## 5 Conclusion

This paper presents a novel technique for continuous non-invasive detection of blood glucose concentration using the direct effect of the blood glucose on the detected speckle patterns collected from covered and uncovered fibers under the magneto-optical effect. The uncovered fiber senses the light tissue interaction, which changes the speckle pattern at the fiber’s output. The covered fiber is not sensitive toward light-tissue interaction, and acquired data at the fiber’s output does not correlate with the blood glucose. The acquired data are transformed using a correlation-based method, which converts the movement of speckle pattern images into 2D movement. The amplitude change over time owing to changes in glucose levels is preprocessed and utilized to train the machine-learning algorithm. The algorithm selection is made for best accuracy by hyper parameter optimization and K-fold validation implementation to prevent overfitting. The Naïve Bayes classification algorithm was selected due to its higher accuracy than other algorithms such as SVMs, neural networks, and KNN. The traditional commercially available blood glucose measurement was used for reference. The results show the possibility of noninvasive blood glucose sensing by an uncovered MM fiber-based sensor under the AC-induced magnetic field. Further development of this technique will allow for the implementation of an automatic and real-time measurement device, which can analyze changes in blood glucose levels suitable for humans.
